# Chronic Khat Use as a Possible Contributor to Advanced Liver Cirrhosis: A Case Report

**DOI:** 10.7759/cureus.104953

**Published:** 2026-03-09

**Authors:** Ahmed A Kassem

**Affiliations:** 1 General Practice, Sheikh Khalifa Bin Zayed Al Nahyan Hospital, Hadibu, YEM

**Keywords:** immune mediated hepatitis, khat and liver disease, khat chewing, liver damage, liver function, liver transplant, portal hypertension, transjugular intrahepatic portosystemic shunt (tips)

## Abstract

We report the case of a 64-year-old man from Yemen with more than 30 years of daily khat (*Catha edulis*) use who developed decompensated liver cirrhosis complicated by portal hypertension, hepatic encephalopathy, and severe pulmonary hypertension suggestive of portopulmonary hypertension. Evaluation, including viral hepatitis serologies and polymerase chain reaction testing, autoimmune markers, and metabolic studies, did not identify an alternative etiology of liver disease. His condition progressed to advanced liver failure, and he was ultimately deemed ineligible for liver transplantation due to severe pulmonary hypertension. This case highlights chronic khat use as a possible contributing factor in advanced liver disease and underscores the importance of considering khat exposure in patients from endemic regions presenting with cryptogenic cirrhosis.

## Introduction

Khat (*Catha edulis*) is a plant whose leaves are traditionally chewed for their stimulant effects, particularly in Yemen, Somalia, and parts of East Africa [1,2]. Khat leaves contain the active alkaloid cathinone, a stimulant structurally related to amphetamines, which has been consistently detected in khat from Yemen and other endemic regions [1,2]. Chronic use, typically daily or near daily over several years and often involving multiple bundles of fresh leaves, may lead to cumulative systemic effects.

Although most research has focused on khat’s psychiatric and cardiovascular effects, emerging evidence suggests a possible association between chronic khat use and liver injury. Reported patterns include hepatocellular injury, cholestatic liver injury, and autoimmune-like hepatitis, with some cases progressing to cirrhosis [3-9]. Khat-associated liver disease remains incompletely understood and is likely underrecognized, particularly in countries where its use is illegal, and patients may not readily disclose consumption [3-5].

Advanced liver disease may also lead to hepatopulmonary complications, including portopulmonary hypertension, characterized by pulmonary arterial hypertension in the setting of portal hypertension, and hepatopulmonary syndrome, which results from intrapulmonary vascular dilatation and impaired oxygenation. These conditions can significantly affect patient outcomes. We present a case of advanced liver cirrhosis with hepatopulmonary complications in a patient with a history of more than 30 years of daily khat chewing, in whom common etiologies of liver disease were excluded. This case highlights a possible association between chronic khat use and advanced liver disease and underscores the importance of considering substance exposures when evaluating patients with cryptogenic liver disease.

## Case presentation

A 64-year-old man from Yemen with a history of daily khat (*C. edulis*) chewing for more than 30 years presented in 2023 with nausea, vomiting, and diffuse abdominal discomfort. In 2023, after becoming aware of reports suggesting potential hepatotoxicity associated with khat use, he voluntarily discontinued chewing and reported no further use thereafter. He denied alcohol consumption or illicit drug use. On physical examination, he had mild scleral icterus, abdominal distension, and bilateral lower extremity edema.

Initial laboratory evaluation demonstrated a mixed hepatocellular and cholestatic pattern of liver injury. Liver enzymes were elevated, including aspartate aminotransferase (AST) (52 U/L), alanine aminotransferase (ALT) (62 U/L), alkaline phosphatase (ALP) (183 U/L), and gamma-glutamyl transferase (GGT) (60 U/L). Hyperbilirubinemia was present with total bilirubin (3.0 mg/dL) and direct bilirubin (2.14 mg/dL). Serum albumin was decreased to 3.1 g/dL, suggesting impaired synthetic liver function. Coagulation studies showed mildly prolonged prothrombin time (PT) (12.8 seconds), while activated partial thromboplastin time (APTT) (33.2 seconds) and international normalized ratio (INR) (1.2) remained within normal limits. Serum ammonia was markedly elevated to 131.3 μmol/L (Table 1).

**Table 1:** Initial laboratory evaluation. AST: aspartate aminotransferase; ALT: alanine aminotransferase; ALP: alkaline phosphatase; GGT: gamma-glutamyl transferase; APTT: activated partial thromboplastin time; PT: prothrombin time; INR: international normalized ratio Symbols used: ↑ indicates values above the normal reference range; ↓ indicates values below the normal reference range

Test	Result	Interpretation	Normal Range
AST	52 U/L	↑	10-40 U/L
ALT	62 U/L	↑	7-56 U/L
ALP	183 U/L	↑	45-120 U/L
GGT	60 U/L	↑	9-48 U/L
Total Bilirubin	3.0 mg/dL	↑	0.1-1.2 mg/dL
Direct Bilirubin	2.14 mg/dL	↑	0-0.3 mg/dL
Albumin	3.1 g/dL	↓	3.5-5.0 g/dL
APTT	33.2 seconds	-	24.0-37.0 seconds
PT	12.8 seconds	↑	9.8-11.7 seconds
INR	1.2	-	0.8-1.2
Ammonia	131.3 µmol/L	↑	11-32 µmol/L
Ferritin	65.1 ng/mL	-	20-300 ng/mL
Alpha-1 Antitrypsin	154 mg/dL	-	101-187 mg/dL
Serum Ceruloplasmin	28 mg/dL	-	20- 50 mg/dL

Evaluation for alternative etiologies of chronic liver disease revealed normal ferritin (65.1 ng/mL), alpha-1 antitrypsin (154 mg/dL), and serum ceruloplasmin level (28 mg/dL) (Table 1). Viral and immunological testing demonstrated no evidence of acute or chronic viral hepatitis. Hepatitis A IgM was nonreactive, and total hepatitis A antibodies were reactive, consistent with prior immunity. Hepatitis B surface antigen was nonreactive, and hepatitis B surface antibody was reactive, indicating immunity. Anti-hepatitis B core immunoglobulin M (anti-HBc IgM) was nonreactive, and hepatitis B virus (HBV) DNA by polymerase chain reaction was undetectable, confirming the absence of active viral replication. Testing for hepatitis C virus antibodies and RNA was negative. Screening for HIV-1 and HIV-2 infection was also negative. Autoimmune evaluation revealed negative antinuclear antibody (ANA), anti-smooth muscle antibody (SMA), and anti-mitochondrial M2 antibody (AMA-M2). Direct antiglobulin test (DAT) was negative, indicating no evidence of autoimmune hemolysis (Table 2).

**Table 2:** Patient viral and immunological test results demonstrating viral exposure and autoimmune status. HAV IgM: hepatitis A virus immunoglobulin M; HAV total AB: hepatitis A total antibody; HBsAg: hepatitis B surface antigen; anti-HBS: hepatitis B surface antibody; anti-HBc IGM: hepatitis B core antibody immunoglobulin M; HBV DNA (PCR): hepatitis B virus DNA by polymerase chain reaction; anti-HCV: hepatitis C virus antibody; HCV RNA (PCR): hepatitis C virus RNA by polymerase chain reaction; HIV: human immunodeficiency virus; ANA: antinuclear antibody; SMA: anti-smooth muscle antibody; AMA-M2: anti-mitochondrial antibody M2 subtype; DAT: direct antiglobulin test

Test	Result	Interpretation
HAV IgM	Non-reactive	No acute Hepatitis A infection
HAV Total Ab	Reactive; immune	Immunity to Hepatitis A (past infection or vaccination)
HBsAg	Non-reactive	No active Hepatitis B infection
Anti-HBs	Reactive; immune	Immunity to Hepatitis B (vaccination or resolved infection)
Anti-HBc IgM	Non-reactive	No recent or acute Hepatitis B infection
HBV DNA (PCR)	Not Detected	No active viral replication
Anti-HCV	Non-reactive	No evidence of Hepatitis C exposure
HCV RNA (PCR)	Not Detected	No active Hepatitis C infection
HIV-1 Antigen	Non-reactive	No early HIV-1 infection detected
HIV-1 Antibody	Non-reactive	No HIV-1 infection
HIV-2 Antibody	Non-reactive	No HIV-2 infection
ANA	Negative	No serologic evidence of systemic autoimmune disease
SMA	15 Units; Negative	Within normal range; no evidence of autoimmune hepatitis
AMA-M2	<20 Units; Negative	No evidence of primary biliary cholangitis
DAT	Negative	No autoimmune hemolysis detected

**Figure 1:**
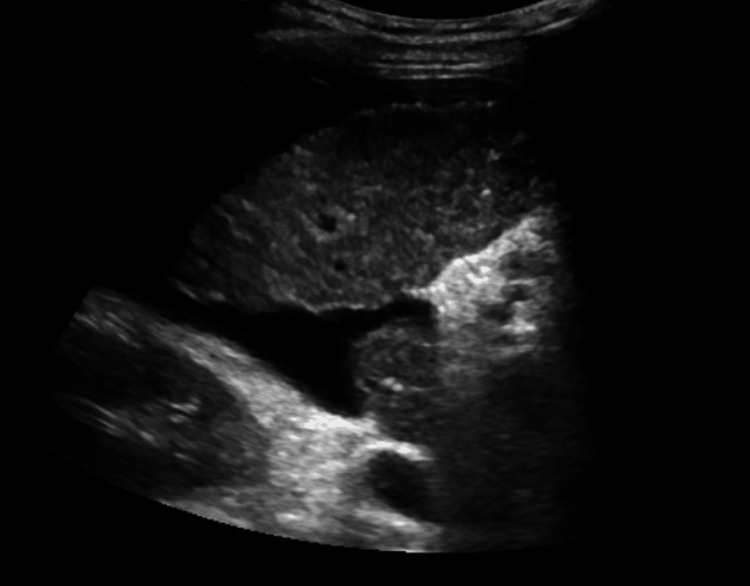
Abdominal ultrasound (transverse view of the right lobe) showing heterogeneous, coarsened hepatic parenchyma with a nodular contour consistent with cirrhosis.

**Figure 2:**
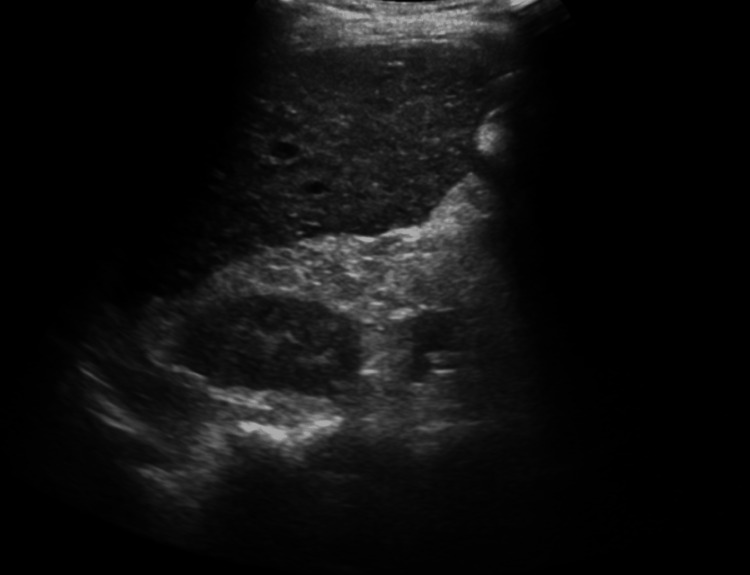
Abdominal ultrasound (sagittal view of the right lobe) showing heterogeneous hepatic parenchyma and a nodular liver contour consistent with cirrhosis.

Abdominal ultrasonography demonstrated a heterogeneous, nodular, and atrophic liver consistent with advanced cirrhosis (Figures 1-2).

**Figure 3:**
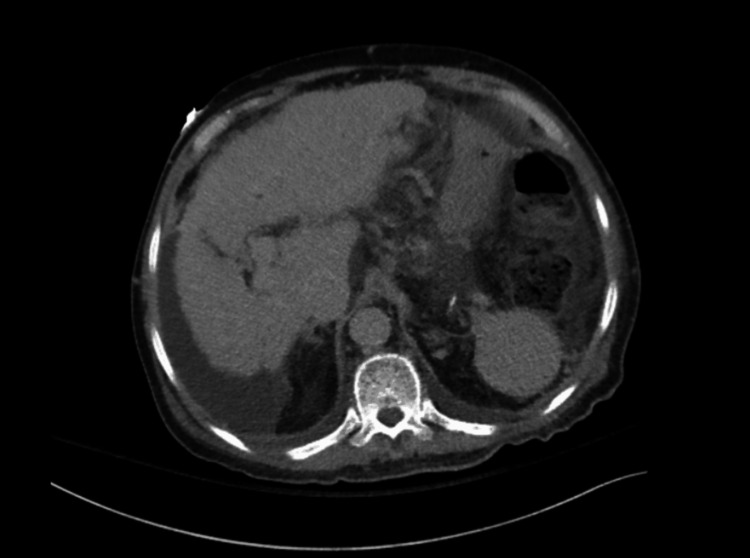
Contrast-enhanced CT of the abdomen and pelvis demonstrating an atrophic liver with surface nodularity consistent with cirrhosis, without intrahepatic bile duct dilation or arterial enhancing hepatic lesions.

Computed tomography of the abdomen and pelvis confirmed cirrhotic morphology (Figure 3).

**Figure 4:**
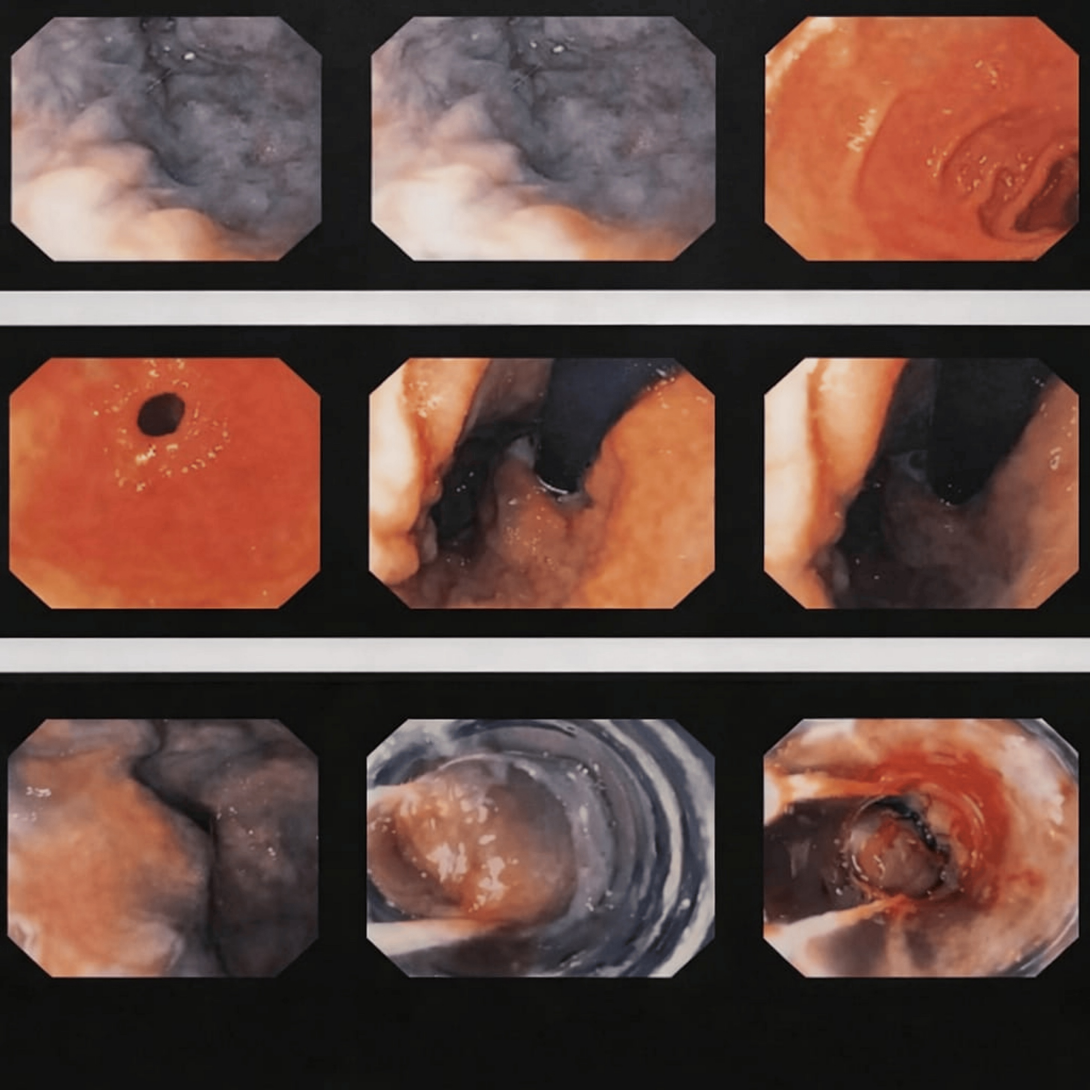
Upper endoscopy demonstrating grade III esophageal varices.

Upper endoscopy revealed grade 3 esophageal varices, which were successfully treated with endoscopic band ligation (Figure 4).

A transjugular liver biopsy was performed, yielding four cores. Histopathological examination demonstrated established cirrhosis with severe bridging fibrosis and regenerative nodule formation. Mixed portal and lobular inflammation, predominantly lymphocytes with occasional plasma cells and neutrophils, was observed. Mild interface activity was present without plasma cell predominance. Focal Mallory hyaline was noted in periportal hepatocytes, and bile duct epithelial cells showed reactive changes without evidence of cholangitis. No significant steatosis was identified. Special stains for iron, copper, and alpha-1 antitrypsin were not documented in the available pathology report. Overall, histopathology confirmed cirrhosis without identifying a specific underlying etiology.

On follow-up evaluation in 2025, laboratory studies demonstrated significant worsening of hepatic function, including markedly elevated AST (473 U/L), ALT (91 U/L), ALP (140 U/L), total bilirubin (13.5 mg/dL), direct bilirubin (6.6 mg/dL), and indirect bilirubin (6.8 mg/dL). Hypoalbuminemia (2.9 g/dL) and low total protein (5.7 g/dL) were also noted. Coagulation studies revealed prolonged PT (22.4 seconds), APTT (45.9 seconds), and INR (1.9), with serum ammonia elevated (47 µmol/L) (Table 3).

**Table 3:** Current liver function, coagulation, and metabolic test results. AST: aspartate aminotransferase; ALT: alanine aminotransferase; ALP: alkaline phosphatase; APTT: activated partial thromboplastin time; PT: prothrombin time; INR: international normalized ratio Symbols used: ↑ indicates values above the normal reference range; ↓ indicates values below the normal reference range.

Test	Result	Interpretation	Normal Range
AST	473 U/L	↑	10-40 U/L
ALT	91 U/L	↑	7-56 U/L
ALP	140 U/L	↑	45-120 U/L
Total Bilirubin	13.5 mg/dL	↑	0.1-1.2 mg/dL
Direct Bilirubin	6.6 mg/dL	↑	0-0.3 mg/dL
Indirect Bilirubin	6.8 mg/dL	↑	<1.3 mg/dL
Total Protein	5.7 g/dL	↓	6.0-8.0 g/dL
Albumin	2.9 g/dL	↓	3.5-5.0 g/dL
APTT	45.9 seconds	↑	24.0-37.0 seconds
PT	22.4 seconds	↑	9.8-11.7 seconds
INR	1.9	↑	0.8-1.2
Ammonia	47 µmol/L	↑	11-32 µmol/L

Hemodynamic assessment via right internal jugular access revealed a hepatic venous pressure gradient (HVPG) of 16 mmHg, consistent with clinically significant portal hypertension. Right-heart catheterization demonstrated elevated pulmonary artery pressures (pulmonary artery systolic pressure (PASP) 66 mmHg) and other hemodynamic measurements, summarized in Table 4.

**Table 4:** Right-heart catheterization and hemodynamic parameters. IVC: inferior vena cava; RA: right atrium; HV: hepatic vein; WHV: wedge hepatic vein; HVG: hepatic venous gradient; RV: right ventricle; PAP: pulmonary artery pressure

Parameter	Result	Normal Range	Interpretation
IVC pressure	0 mmHg	0-8 mmHg	Normal
RA pressure	0 mmHg	2-8 mmHg	Low-normal
Free HV pressure	0 mmHg	2-8 mmHg	Low-normal
WHV pressure	16 mmHg	4-10 mmHg	Elevated
HVG	16 mmHg	<5 mmHg	Elevated (portal hypertension)
RV pressure	60/1 mmHg	15-30/2-8 mmHg	Elevated systolic pressure
PAP	66/2 mmHg	15-30/8-15 mmHg	Elevated (portopulmonary hypertension)

These findings are consistent with portopulmonary hypertension. Given the markedly elevated pulmonary pressures and concern for right ventricular strain, transjugular intrahepatic portosystemic shunt (TIPS) placement was considered high risk and was not pursued.

During hospitalization between 2024 and 2025, the patient experienced multiple complications of decompensated cirrhosis, including hepatic encephalopathy, ascites, variceal bleeding, right-sided heart failure, protein-calorie malnutrition, hyponatremia, aspiration pneumonia, and septic shock secondary to extended-spectrum beta-lactamase (ESBL)-producing bacteria.

Ongoing management included supportive therapy with diuretics (furosemide and spironolactone) and lactulose for hepatic encephalopathy, along with ciprofloxacin prophylaxis for spontaneous bacterial peritonitis. Additional measures included omega-3 supplementation, sildenafil, and tailored nutritional support.

By January 2025, the patient was evaluated for liver transplantation; however, the procedure was declined due to severe pulmonary hypertension, which significantly increased perioperative risk.

## Discussion

Chronic khat chewing, derived from *C. edulis*, is prevalent in Yemen and parts of East Africa but remains underrecognized as a potential contributor to liver disease in Western healthcare settings. Khat leaves contain the active alkaloid cathinone, a stimulant structurally related to amphetamines, which has been consistently detected in khat from Yemen and other endemic regions [1,2]. This may be partly due to underreporting by patients, particularly in countries where khat is restricted or illegal, limiting clinician recognition of khat-associated liver injury [3-5]. Increasing evidence has described an association between long-term khat consumption and hepatotoxicity, with reported manifestations ranging from asymptomatic elevations in liver enzymes to chronic hepatitis, fibrosis, and cirrhosis [3-9]. Specifically, studies in chronic khat chewers have documented elevations in liver enzymes, supporting a potential hepatotoxic effect of cathinone [2,3].

The mechanisms underlying khat-associated liver injury are not fully understood. Proposed mechanisms include oxidative stress, mitochondrial injury related to cathinone, and immune-mediated hepatocellular damage that may lead to progressive fibrosis [3,4]. In addition, contaminants such as pesticides or heavy metals present in khat leaves have been proposed as potential contributing factors to hepatotoxicity [5].

In the present case, extensive evaluation excluded viral hepatitis, autoimmune liver disease, metabolic disorders, alcohol-related liver injury, and other common etiologies of cirrhosis. The patient reported a history of more than 30 years of daily khat chewing, and in the absence of alternative explanations, the clinical presentation is consistent with khat-associated liver injury as a possible contributing factor to the development of advanced cirrhosis. However, because this report describes a single case, a direct causal relationship cannot be definitively established.

When evaluating suspected toxin- or drug-related liver injury, structured causality assessment tools such as the Roussel Uclaf Causality Assessment Method (RUCAM) are commonly used to estimate the likelihood of hepatotoxicity by incorporating clinical, laboratory, and exposure data [4]. Although complete information required for formal scoring was not available in this case, the patient’s prolonged exposure history, exclusion of other etiologies, and previously reported associations in the literature support a possible relationship between chronic khat use and the patient’s liver disease.

Notably, the patient also developed severe pulmonary hypertension with features suggestive of portopulmonary hypertension, a recognized complication of advanced portal hypertension and cirrhosis. This complication ultimately contributed to the patient being deemed ineligible for liver transplantation.

This case highlights the importance of clinicians considering khat exposure when evaluating patients from endemic regions who present with cryptogenic liver disease. Increased awareness is particularly relevant in Western healthcare settings where khat use may not be routinely screened for during clinical evaluation. Further clinical and epidemiological studies are needed to clarify the potential hepatotoxic mechanisms of chronic khat exposure, identify risk factors for disease progression, and better define its long-term hepatic and systemic effects [1-5,7,9].

## Conclusions

Chronic khat (*C. edulis*) use should be considered a possible contributor to unexplained liver cirrhosis, particularly in patients from regions where khat chewing is common but may not be routinely disclosed in countries where the substance is restricted. In this patient, extensive evaluation excluded viral, autoimmune, metabolic, and alcohol-related causes of liver disease, supporting chronic khat exposure as a potential contributing factor. Because causality cannot be established from a single case report, further clinical and epidemiological studies are needed to clarify the relationship between chronic khat use and liver injury, as well as its potential systemic effects.
